# Immunology and individuality

**DOI:** 10.7554/eLife.47384

**Published:** 2019-04-05

**Authors:** Thomas Pradeu

**Affiliations:** 1 ImmunoConcept CNRSUniversité de BordeauxBordeauxFrance; 2 Institut d'histoire et de philosophie des sciences et des techniquesUniversité Paris 1 Panthéon SorbonneParisFrance

**Keywords:** immunogenicity, Individuality, Philosophy of Biology, Philosophy of Science, self, nonself

## Abstract

Immunology and philosophy have a rich history of dialogue. Immunologists have long been influenced by ideas from philosophy, notably the concept of 'self', and many philosophers have explored the conceptual, theoretical and methodological foundations of immunology. Here, I discuss two aspects of this dialogue: biological individuality and immunogenicity.

What do philosophers say about immunology, and to what extent can this be useful to immunologists? One remarkable feature of modern immunology is that it uses a vocabulary that has been strongly influenced by philosophy. The philosophical concepts of 'self' and 'nonself', in particular, have played a central role in immunology since the 1940s. Reflecting on this vocabulary enables us to better understand why it was adopted, what its underlying assumptions are, and whether it should be maintained or revised in light of what we know about immunology today.

In this article I show how a philosophical approach can shed light on two key aspects of current immunology. The first is biological individuality: what defines the unity, boundaries, uniqueness and persistence of a living thing according to immunology, especially in the context of what we are learning about the interactions between microbiota and the immune system? The second aspect is immunogenicity: that is, the ability of certain entities to trigger an effector immune response that destroys a target.

## Immunological individuality

Our fascination with biological individuality dates back to Aristotle, possibly earlier, and has been a central issue in immunology from the end of the 19^th^ century (Medawar, 1957; Richet, 1894). The fundamental question raised by reflections on biological individuality is what makes a living being a cohesive, relatively well-delineated and often unique entity that remains the 'same' through time despite undergoing constant change (Santelices, 1999; West et al., 2015). Biological individuality is relative in that it depends on the question being asked. Moreover, it comes in degrees, in so far that the four main elements of biological individuality – cohesion, delineation, uniqueness and persistence – can be expressed to different levels in a living being (Pradeu, 2016; Santelices, 1999).

Though immunology is not the only scientific field addressing the issue of biological individuality, it does make a major contribution to this question. The immune system plays a key role in monitoring every part of the organism and maintaining the cohesion between the components of that organism, making each individual unique and constantly re-establishing the boundaries between the organism and its environment (Pradeu, 2012).

The question of the self-nonself is particularly relevant in defining biological individuality and has been strongly shaped by the Australian virologist Macfarlane Burnet (Tauber, 1994). His conceptual and theoretical reflections on the self and not-self (inspired by philosophy) were later adopted by a vast majority of immunologists. Burnet suggested that every entity that is foreign to the organism is rejected by the immune system, while every entity that originates from that organism does not trigger an immune response (Burnet, 1969). This framework made it possible to account for various immune responses, from pathogens to grafts.

Burnet considered immunology to be more a philosophical problem, rather than a scientific one, and was strongly influenced by the mathematician and philosopher Alfred North Whitehead, who had given the notion of the self a central role in his philosophy (Anderson and Mackay, 2014). The dialogue between immunologists and philosophers has continued ever since: immunologists borrow concepts from philosophy, especially when they reflect on the issue of individuality, and reciprocally, many philosophers use immunology as a major source of inspiration (Cohen, 2009).

Philosophers have improved our understanding of how the conceptual framework of the self-nonself was built, and helped put into question its theoretical and empirical foundations (Pradeu, 2012; Swiatczak and Rescigno, 2012; Tauber, 1994). Scientific data collected since the 1990 s have revealed that the immune system also responds to endogenous components, that is, to the self. In fact, a significant degree of autoreactivity and autoimmunity is indispensable for a healthy immune system. Immune responses such as the phagocytosis of dead cells, tissue repair and regulatory responses are in most cases responses to the self (Rankin and Artis, 2018). Moreover, it is now clear that many foreign entities, such as microbial communities (known as microbiota), are actively tolerated by the immune system rather than eliminated (Chu and Mazmanian, 2013).

**Figure 1.**
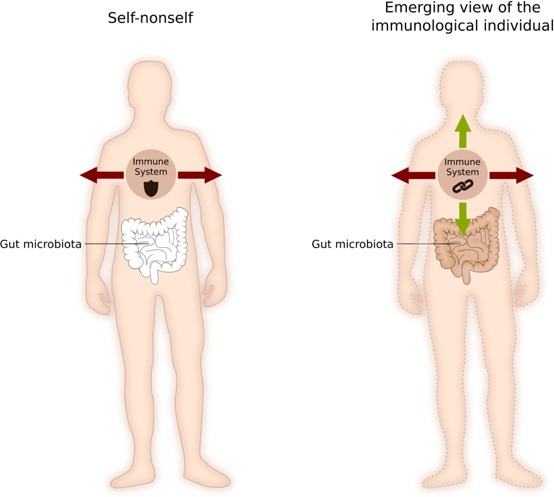
How immunology defines a biological individual. According to the 'self-nonself' framework (left), the immune system is mainly a system for targeting and killing foreign bodies. Interfaces, such as the gut lumen, belong to the ‘outside’ of the organism, and boundaries are strict and fixed. According to the newly emerging 'immunological individual' framework (right), the immune system can eliminate self and nonself elements, it can tolerate self and nonself elements, but it also reinforces the cohesion between bodily constituents. In this framework, boundaries are constantly being redefined by the action of the immune system. Image credit: Wiebke Bretting (CC BY 4.0).

These developments have led many to conclude that the self-nonself framework should be revisited, and that we should switch from an internalist view (which sees the individual as insular, autonomous and endogenously built) to a more interactionist view (which sees an organism as an ecosystem that is constantly interacting with its environment; McFall-Ngai et al., 2013). Although biological individuality remains a key question in immunology, the way that scientists see it has changed: the notion of self-nonself has evolved to the idea of an individual made of heterogeneous elements with constantly redefined boundaries, in which the immune system can not only eliminate, but also actively tolerate the elements with which it interacts (Figure 1; Pradeu, 2012).

## Immunogenicity

The self-nonself framework also offers an explanation of immunogenicity: that is, how an effector immune response (a response leading to the elimination or the neutralization of a target) is activated. Some hypotheses, such as the 'danger theory', suggest that the immune system does not distinguish between self and nonself: rather, it differentiates between things that cause damage and those that do not (Matzinger, 1994). However, together with fellow immunologists, I suggest another alternative: the discontinuity theory of immunity (Pradeu et al., 2013; Pradeu and Carosella, 2006).

The discontinuity theory proposes that effector immune responses are triggered by sudden changes in the molecular motifs that interact with the receptors of the immune system (e.g., pattern-recognition receptors, NK cell receptors, B cell receptors, T cell receptors and cytokine receptors). In contrast, a persistent motif, or a slowly appearing one, do not trigger an effector response, rather, it leads to a tolerogenic immune response (where a target will be accepted rather than eliminated). Space and time are important in the discontinuity theory: the vast majority of immune responses occur in tissues rather than in the blood, and different tissues have different baseline levels of immune activation, so any theory of the immune response must account for the tissue-specific nature of the response.

An immune response may be due to all sorts of sudden changes, and could be related to motifs being recognized and/or changes to the immune system (such as the migration of motifs or immune cells from one tissue to another, or the rapid appearance of a pathogen or a tumour). What clearly distinguishes the discontinuity theory from the self-nonself theory is that the criterion of immunogenicity is not the origin of the antigen (as it is in the self-nonself framework): rather, it is the speed of change in the relevant tissue (Table 1). Therefore, persistent or slowly appearing 'nonself motifs' are tolerated by the immune system, while fast-appearing 'self motifs' cause an effector response.

**Table 1.** Different theories of immunogenicity. The self-nonself theory and the discontinuity theory of immunity predict the same outcomes for persistent or slowly changing endogenous (self) elements, and also for suddenly appearing and/or rapidly changing exogenous (nonself) elements. The theories make different predictions for rapidly changing endogenous elements, and for persistent or slowly changing exogenous elements.

Motifs	Examples	Self-nonself theory	Discontinuity theory
Rapidly changing endogenous elements	- Some significant bodily transformations, when uncontrolled (e.g., puberty, metamorphosis, pregnancy)	tolerogenic response	effector response
Persistent or slowly changing endogenous elements	- Usual functioning of the body	tolerogenic response	tolerogenic response
Persistent or slowly changing exogenous elements	- Many components of the microbiota acquired early during ontogeny - Chronic viruses	effector response	tolerogenic response
Suddenly appearing and/or rapidly changing exogenous elements	- Microorganisms that invade the organism suddenly - Most grafts	effector response	effector response

This could be relevant for the field of onco-immunology (Ribas and Wolchok, 2018; Pauken and Wherry, 2015). For example, the discontinuity theory predicts that a slowly growing tumour triggers a tolerogenic immune response, whereas a tumour that is growing rapidly (or a tumour in a microenvironment that is changing rapidly) triggers an effector response. The discontinuity theory has also been used to shed light on a range of different topics, including the effects of chemotherapeutic agents on immunomodulation in cancer (Hodge et al., 2013), the constant 'education' of natural killer cells to ensure tolerance of bodily constituents (Boudreau and Hsu, 2018), repeated vaccinations in immunocompromised individuals (Rinaldi et al., 2014), and mathematical models of immune activation (Sontag, 2017). Depending on future experimental results, this theory will be enriched, revised or, perhaps, abandoned.

## Conclusion

Immunology is one of the most theoretical and most philosophical fields within the life sciences, and the ongoing dialogues between immunologists and philosophers are likely to continue. The list of questions worth discussing include the following: i) How can we combine the various types and levels of explanation in immunology (from molecules to system) into an integrative framework? ii) Which principles should one use to offer satisfactory and fruitful classifications of immune components (Mantovani, 2016)? iii) How can immunology be enriched by contributions from other areas of biology and beyond (including physics and computer science?) iv) Can one define immunity and immunology? It is now clear that the immune system does many things besides defending against pathogens: for example, it is involved in development and tissue repair. As the field of immunology has become broader (to the extent that it overlaps with many areas of physiology), its boundaries have become blurred (Rankin and Artis, 2018; Truchetet and Pradeu, 2018). For these challenges and many others, a close alliance between philosophers, biologists and physicians seems full of promise.

## Note

This Feature Article is part of the Philosophy of Biology collection.
